# Using patients’ experiences to identify priorities for quality improvement in breast cancer care: patient narratives, surveys or both?

**DOI:** 10.1186/1472-6963-12-271

**Published:** 2012-08-22

**Authors:** Vicki Tsianakas, Jill Maben, Theresa Wiseman, Glenn Robert, Alison Richardson, Peter Madden, Mairead Griffin, Elizabeth A Davies

**Affiliations:** 1 Florence Nightingale School of Nursing and Midwifery, King’s College London, 57 Waterloo Rd, London SE1 8WA, UK; 2 The Royal Marsden NHS Foundation Trust, Fulham Road, London SE3 6JJ, UK; 3 School of Health Sciences University of Southampton & Southampton University Hospitals Trust, Highfield, Southampton, SO17 1BJ, UK; 4 Thames Cancer Registry, King’s College London, 42 Weston Street, London SE1 3QD, UK; 5 Guys and St Thomas’ NHS Foundation Trust, St Thomas’ Street, London SE1 9RT, UK

**Keywords:** Experience-Based Co-Design, Patient questionnaire, Survey, Quality improvement, Patient experience, Breast cancer, Narrative interviews

## Abstract

**Background:**

Patients’ experiences have become central to assessing the performance of healthcare systems worldwide and are increasingly being used to inform quality improvement processes. This paper explores the relative value of surveys and detailed patient narratives in identifying priorities for improving breast cancer services as part of a quality improvement process.

**Methods:**

One dataset was collected using a narrative interview approach, (n = 13) and the other using a postal survey (n = 82). Datasets were analyzed separately and then compared to determine whether similar priorities for improving patient experiences were identified.

**Results:**

There were both similarities and differences in the improvement priorities arising from each approach. *Day surgery* was specifically identified as a priority in the narrative dataset but included in the survey recommendations only as part of a broader priority around improving *inpatient experience*. Both datasets identified *appointment systems*, *patients spending enough time with staff*, *information about treatment and side effects* and more information at the end of treatment as priorities. The specific priorities identified by the narrative interviews commonly related to ‘relational’ aspects of patient experience. Those identified by the survey typically related to more ‘functional’ aspects and were not always sufficiently detailed to identify specific improvement actions.

**Conclusions:**

Our analysis suggests that whilst local survey data may act as a screening tool to identify potential problems within the breast cancer service, they do not always provide sufficient detail of what to do to improve that service. These findings may have wider applicability in other services. We recommend using an initial preliminary survey, with better use of survey open comments, followed by an in-depth qualitative analysis to help deliver improvements to relational and functional aspects of patient experience.

## Background

Patients’ experiences have become increasingly central to assessing the performance of healthcare systems worldwide [1]. It is now common to judge quality of care not only by measuring clinical quality and safety but also by gathering the views of patients [2-4]. These can be used as part of systems for developing policy, monitoring the performance of health care organizations, [5] for informing patient choice and making healthcare organizations more transparent and accountable to their local populations, [6] and as a mechanism for improving patient experiences in specific local services [7].

The relative value of assessing patient experience using quantitative and qualitative methods remains the subject of ongoing debate. Simple measures of “satisfaction” are now recognized as being too greatly influenced by prior expectations [8] and of providing little specific information that can be used in quality improvement [9-11]. The most commonly used patient experience surveys in the US and English health care systems [12,13] have been developed using data from interviews and focus groups with patients and staff and aim to measure how specific aspects of care were experienced by patients. A potential strength of such measures is they can compare experiences of different patient groups in different services (or organizations) and monitor changes over time. However, many surveys focusing on experience are still commonly referred to as “satisfaction surveys” and users of survey data do not always distinguish sufficiently clearly between these two concepts [13-15].

In contrast, collecting data on patient experience using in-depth qualitative interviews can elicit a detailed multi-faceted understanding of the meanings individuals attach to specific elements of their care. This can be helpful given that a good patient experience is about both ‘the what’ (transaction) and ‘the how’ (relational) of interaction with providers [16]. The usefulness of patient stories lie in their ability to ‘communicate vividly the multi-layered texture and complexity of experience in hospital, its intensity and human experience’ [16]. However, patient narratives can be perceived as time-consuming to collect and neither representative nor generalisable. Regardless of which data collection methods are used, a co-ordinated strategy is required to use patient feedback to improve services and evaluate any subsequent changes.

This paper compares two independently collected datasets of patient experiences in the same breast cancer service generated through two different methods (Experience-Based Co-Design [EBCD] and a patient experience survey) and the recommendations for quality improvement that emerged from each. The first dataset was derived from a narrative-based approach to gathering patient experiences as an integral part of a planned change process (EBCD), and the second from an anonymous postal cancer patient experience survey undertaken contemporaneously. To date, there has been no direct comparison of data and improvement priorities from a narrative-based approach, such as EBCD, with those arising from a patient experience survey in the same service. The aims of the comparison are: a) to determine whether the two methods identified similar improvement priorities as part of a quality improvement process in terms of patients’ experiences of care and (b) to consider potential strengths and weaknesses of each method. This paper does not report on the actual subsequent quality improvement work or its outcomes as this is the subject of an ongoing study. Our data and analysis is confined to a single breast cancer service but is likely to have wider applicability.

## Methods

### Setting

The EBCD and patient survey were part of a project designed to improve patient experience in the breast cancer services of an Integrated Cancer Centre, spanning two teaching hospitals (sites A and B) in a large multi-cultural city in the UK. The two approaches were used independently to collect, analyze data and feedback findings to the two services. The same breast cancer teams cared for the EBCD and survey participants though care pathways and therefore experiences in each hospital were likely to differ. Hospital A was a radiotherapy centre receiving referrals from a large area while hospital B provided a screening service and referred some patients to Hospital A. The National Research Ethics Service advised that the EBCD and the survey were service evaluation and approval from them was not necessary. All patients and staff were given information sheets and asked to sign a consent form before interviews took place. Local Research and Development approval was obtained from each hospital. We conformed to ethical principles during the design and conduct of the study.

### Experience-Based Co-Design approach

The EBCD approach is described in detail elsewhere [11,17] but essential components include the use of filmed patient narratives, ethnographic observation and interviews with staff as part of a planned change process. Patient narratives are used to capture and understand patient experiences of the care pathway and to identify ‘touchpoints’ along their journey - the crucial moments, good and bad that shape a patient’s overall experience. The concept of ‘touchpoints’ originated in the airline industry and has been defined as the key moments or moments of truth where people come into contact with a service (in our case a breast cancer service) and where their subjective experience is shaped [11]. At interview, patients were invited to ‘tell their story’, describing their experiences of care since first diagnosis. Interviews were unstructured, however, a broad topic guide was used to assist the researcher. Observation and staff interviews were undertaken as an integral part of a wider quality improvement project, but these data are not the subject of this paper. The 23 patients (13 at Hospital A and 10 at Hospital B) who shared their narratives were recruited by clinical nurse specialists (CNSs) at each hospital site who oriented patients to the overall aim of the study and the interview subject area. All patients were nearing or at the end of treatment for breast cancer and were judged well enough by the CNS to participate. Filmed, narrative interviews were scheduled with consenting patients, carried out by an experienced qualitative researcher (VT), and lasted between 1–3 hours. Audio recordings of the narrative interviews were transcribed verbatim.

There were two purposes to conducting narrative interviews and therefore two phases of analysis. The primary purpose was to gather narratives to enable production of a compilation film illustrating the key ‘touchpoints’ experienced by patients along the way. The second purpose of conducting narrative interviews was to identify key touchpoints for comparison with the survey data. This meant a second set of analysis was conducted (see section on comparison of areas for quality improvement).

The production of the compilation film began with two experienced qualitative researchers (VT, anthropologist and TW, nurse and social scientist) viewing the films independently to ensure analytical rigour. Researchers identified recurring themes (both positive and negative) that shaped patients’ overall experiences, identified here as ‘touchpoints’. VT and TW compared and contrasted their understanding of significant ‘touchpoints’ and reached consensus based upon recurrence of themes within narratives. For example, almost all breast cancer patients experienced problems with ‘day surgery’. As a result, day surgery was identified as a ‘touchpoint’, as it shaped patients’ overall experience with the service. Individual filmed interviews were returned to patients to ensure they were happy for parts of their interview to be used in the compilation film. Filmed interviews were then edited and compiled into one short film depicting key (positive and negative) ‘touchpoints’ experienced by patients along the way. VT and TW selected the most compelling film clips to depict each touchpoint. This film was viewed by patients at a patient event and by both staff and patients at a co-design event.

**Figure 1:**
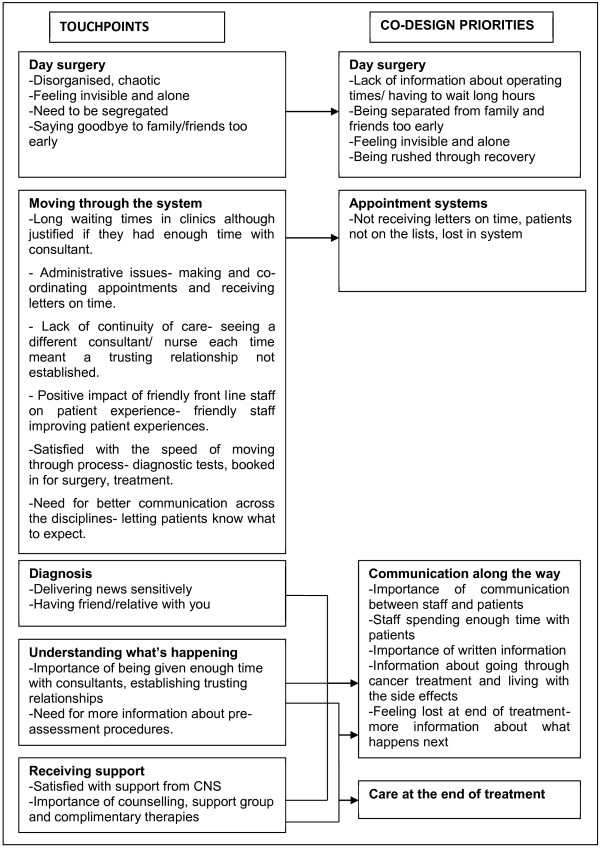
Process by which ‘touchpoints’ became ‘co-design priorities’ *Chemotherapy and radiotherapy were also identified as ‘touchpoints’ but were not identified as co-design priorities and therefore not included in this paper. Being an inpatient was also not included although it was identified as a priority in the survey recommendations.

EBCD priorities for improvement were identified by both patients and staff during the co-design event as a result of discussion generated through the patient film. Initially, key issues for improvement were identified independently at the patient and staff events. Many of the ‘touchpoints’ emerging from narrative interviews were identified as priorities but were modified through discussion at the co-design event (see Figure 1) to become four co-design priority areas for improvement. These discussions involved patients and staff working together to agree on priorities with a focus on patient-centred care [18].

### Patient experience survey approach

A patient experience survey designed and validated by the Picker Institute [19,20] for use at another Cancer Centre in the same city was adapted and piloted for this study. The adaptation and piloting is described elsewhere [21] and involved consulting staff and patients to ensure the content of the questionnaire was understandable to patients, that it focused on topic areas perceived as relevant to patients (content validity) and provided specific information useful for quality improvement at each hospital site. One hundred and two questions were selected and organized into six sections: *Finding out what was wrong with you, Outpatient treatment and appointments*, *Recent overnight or day case stay*, *Leaving hospital*, *Clinical nurse specialist care* and *Overall views about care*. Two final open questions asked patients what they thought was particularly good about their care and what areas they thought could be improved.

One hundred and twenty seven breast cancer patients were identified by administrative staff using hospital systems at site A, while a smaller sample of 37 patients was selected by one clinician from a clinical breast cancer database at site B. All patients had received a breast cancer diagnosis and were nearing or at the end of treatment. Patients received a questionnaire by post, an information sheet and a letter from the lead cancer nurse explaining the survey was voluntary and confidential, and that results would be used to improve services. Return of the survey was taken as consent to participate. Sixty five per cent (82/127) of breast patients approached at site A returned completed questionnaires and 70% (26/37) at site B. A high proportion of patients (66%, 54/82 at site A and 85%, 22/26 at site B) added comments about their care and suggestions for improving services. The demographic and clinical characteristics of patients taking part and factors associated with their response are described elsewhere [21]. Fewer patients were identified at site B for the survey than routine cancer registration data had suggested and on which we had based a comparative survey study with site A. In general survey samples should be representative of the population of interest. Techniques for minimizing selection bias include using a specific time period as a sampling frame, taking a random sample of all patients, or sometimes in hospitals that see and treat smaller numbers overall, recruiting all available patients. The size of a sample may be pragmatically determined by financial constraints, staff time for recruitment and by sample size calculations where a specific improvement goal from previous patient experience data can be formulated.

Although we had some doubts about the representativeness of the sample of patients at hospital B, separate feedback reports were produced describing the results as percentages of experience reported at site A and B for each area of care where at least twenty patients responded to a question. Results were also presented as bar charts. A thematic, qualitative analysis of responses to open ended questions was undertaken by ED and reported with examples. Recommendations for areas of service improvement were identified after data collection and analysis were complete by AR, PM and ED when less than 75% (or more than one in four) patients reported a less good experience. This threshold was a pragmatic one, which followed the earlier use of other survey results in London [20]. The report was provided to the Centre’s lead clinicians and the project management team where the results were discussed, and fed back by MG to the multi-disciplinary teams at each site. These data were not fed back or discussed as part of the EBCD process.

### Comparison of areas for quality improvement

For the purposes of identifying key themes/touchpoints for comparison with survey data, interview transcripts were categorized, tabulated and thematically coded by touchpoint by VT and experienced qualitative researcher, JM (nurse, social scientist). Findings from the qualitative data were tabulated alongside the main survey findings to determine whether the two datasets identified similar issues about patients’ experiences. To determine whether the comments made in the survey were of a similar nature to the more extensive descriptions within the filmed narratives, VT carried out a thematic analysis of all the questionnaire comments and compared these to the ‘touchpoint’ analyses from the patient narratives.

VT and a researcher experienced in quantitative and qualitative analysis (ED) then together compared and contrasted the EBCD and survey data sets for each narrative ‘touchpoint’ and issue identified in the survey. Issues identified by one dataset but not the other were also noted as well as those where the findings seemed similar. The priorities for improvement identified by each method were also tabulated and compared.

The data from 10 patient interviews at hospital site B provided a rich picture of experience at that site and provided enough participants to subsequently identify improvement priorities at the co-design events. However, the number of patients responding to the survey at site B (n = 26) or for whom all questions were relevant was too small to calculate meaningful summary percentages and the number and range of open comments was limited. Overall, the lower than expected number of patients provided at site B for the survey and uncertainty about their selection, and therefore representativeness, led us to decide that the survey data could not support further comparative analyses of the different care pathways. These data from both interviews and surveys at site B are therefore excluded from this paper which focuses solely on reporting the comparison for hospital A (13 interviews and 82 survey responses).

## Results

In this section, we compare (a) qualitative themes (‘touchpoints’ that arose from the EBCD patient narratives) and (b) key findings from the survey in the same areas. We then compare the priorities/recommendations for improvement that arose from each method.

### Receiving a diagnosis

A high proportion of survey respondents reported they had been given their diagnosis with sensitivity and care (93%, 75/81) and had completely understood the explanation (79%, 63/81); no respondents added comments about diagnosis. In contrast, patient narratives described mixed experiences of how the diagnosis was communicated. Some patients were pleased with the hospital doctor’s use of drawings, simple language or reassuring style:

*"He had to deliver some pretty horrible news. …I had a lot of faith in him. He explained things to me. He did a wonderful thing, he actually did little drawings of the things for me. Actually drawing what was going to happen was great."* (P3)

Others felt the diagnosis was delivered insensitively:

*"When I was told about the extra tumour I had that had gone into the lymph nodes, I have to say he’s a wonderful man and a wonderful surgeon, but I don’t really think he had the bedside manner. He came out with all these long words, and I just sat and looked at him. I think the telling of the news could have been given in a better way."* (P2)

The narratives revealed all patients felt it important to have a relative or friend with them at diagnosis to give support and take in information, but not all patients had someone with them. The survey data showed that around half (56%, 46/82) of patients when asked ‘who else was present when you were told what was wrong with you?’ reported that a relative or friend had been with them. However, overall when asked ‘did you want somebody else to be present,’ 39% (32/82) answered that they would not have wanted anyone else there. Since 31% (10/32) of this group had reported a relative or friend with them, this suggests some ambiguity with the question. The second question either caused confusion about who any additional persons might have been, or that the remaining 22 patients (one quarter of the sample of 82) had not in fact wished anybody else to be present.

### Having day surgery

The EBCD patient narratives showed almost all patients had negative experiences of day surgery; describing it as fragmented service, experienced as disorganized and chaotic. Patients spoke about feeling invisible and alone and neglected by staff. Particular issues in day surgery included being separated from family and friends in the waiting area too early and mixed sex facilities being unacceptable for women undergoing breast surgery. Two patients spoke about their experiences in day surgery:

*"I did ask where my timeslot was, and they said they didn’t know. I may as well have been invisible. I felt as though throughout this journey, I was important, all the tests I had were important, I was made to feel important, but on the day of my operation, except for the consultant and the teams in the theatres who were brilliant, I felt like just any other Joe waiting to have an operation. And that was awful…I didn’t want preferential treatment; I just wanted to feel like a person, a human being."* (P6)

Day surgery was revealed as one of the major touchpoints in the EBCD narratives, By comparison the survey results were not reported specifically for day surgery but for all in-patient and day case stays. However, of the 15 patients who reported a day case stay, three added comments about day surgery being chaotic and poor staff attitudes.

### Being an inpatient

The majority of patients interviewed described negative experiences on one particular ward. They talked about feeling neglected and receiving inadequate nursing care that did not seem geared towards care for cancer patients. Patients felt that staff lacked the knowledge and skills and the positive attitude to care for them. Many patients felt they were not treated with respect and dignity; they were treated as a number rather than a person. Some patients felt that visiting family and friends were not welcomed by staff. There was also a lack of timely assistance from staff with wound dressing, bed pans and bathing. Experience of being an inpatient was identified as a key ‘touchpoint’ for patients in the EBCD narratives:

*" I felt that there was just a real lack of basic respect. Really small things, like looking at you and making eye contact with you and speaking to you and treating you as a human being, were often lacking. There were some nursing staff who were fantastic and did all of that, but there were quite a lot who didn’t, and it made the experience a really negative one. What I did after a while was I didn’t really give myself morphine when I wanted it on the pump because I became too scared of what was going to happen if I wasn’t fully in control… I don’t think that helped my recovery."* (P1)

The survey data did not ask patients which ward they attended, but three specific additional comments about inpatient care described negative experiences on the same ward. Overall, a high proportion of patients in the survey data reported feeling confident in the doctors (80%, 41/51) and being treated with respect and dignity by doctors (80%, 41/51) and nurses (80%, 42/52) in hospital. However, patients less commonly reported understanding side-effects (69%, 34/52), family and friends being involved in treatment decisions (47%, 25/52), being given written information about their condition or treatment (60%, 32/53) or enough nurses being available (67%, 34/51). Over one half of patients (58%, 30/52) reported being in pain or discomfort some of the time and only a small proportion (29%, 15/52) that staff had discussed additional support they might need to resume usual activities.

### Moving through the system

Patient narratives revealed all patients were satisfied with the speed of moving through the system with tests and treatments being efficiently arranged. Additional comments in the survey data identified speed of moving through the system as important, although efficiency was not addressed specifically in the questionnaire. Long waiting times in clinics were identified as stressful in the patient narratives, but most felt waits were justified if others needed more time with consultants. One patient reflected on her experience:

*" The waits are very long, but it didn’t bother me in the slightest because I knew that when I was in there, they would give me whatever time I needed. It didn’t matter at all. I know there were people in the waiting room moaning and groaning about having to wait, but actually, you might have to wait, but they did take their time with you. They never, ever rushed you in and out."* (P5)

Appointment systems were identified as a co-design priority area for improvement. The majority of patients had negative experiences with administrative processes, for example, with receiving appointment letters or making appointments. One patient spoke of her experience:

*" Not always did I have my appointments when I was supposed to have them. If I didn’t keep a check and ring - I am a bit tenacious like that – I would have missed appointments and check-ups."* (P2)

Patients were not questioned about clinic waiting times in the survey but this issue was identified by 12 comments, as were administrative problems (5 comments).

Patients stressed the importance of continuity of care by particular staff and the importance of building trust. This was identified as a major ‘touchpoint’ for patients. In the survey, 83% (68/82) of patients agreed that ‘people were working closely together all, or most of the time’; communication between the departments was not specifically addressed and continuity of care was not covered at all.

### Understanding what’s happening

Patient narratives showed that overall patients were highly satisfied with the care from consultants and the time they spent with them. Communication and information along the way was important to patients and identified as one of the co-design areas for improvement. One patient talked about the importance of spending time with consultants:

*" I did see an oncologist, and a radiotherapist, and they spent hours, I mean literally hours, with us talking about the drug treatment that I was going to have, the radiotherapy, whether to have radiotherapy on both sides, and I was really impressed with that. I never felt that they were under pressure, and they gave us all the information we could possibly want. So you felt that time was absolutely not a question, that if you had things that you needed to ask, things that you needed to know somebody was there for you."* (P4)

This helped build a sense of trust. However, the majority also raised in their narratives the need for better communication across different departments about what would be happening to them, and typically they felt they needed more information about pre-assessment procedures and care for the wound at home after surgery. One patient described her experience of pre-assessment procedures:

*" The pre-procedures weren’t explained. I wasn’t told I was going to have for breakfast - at 8:00 in the morning - a giant needle syringe full of blue radiation injected into my nipple [laughs] without any painkiller. A lot of the things are quite brutal and you’re not told they’re going to happen. It’s just like, ‘Now we’re going to do this to you,’ and you do begin to feel humiliated because you’re constantly naked and having horrible things done, injections and poked around. You feel like you’re a bit of meat on a conveyor belt."* (P3)

A lack of information about procedures negatively impacted patient’s experience with the service as they felt ill-prepared to deal with what was happening to them.

The survey data showed that the majority of patients (80% or more) felt they spent enough time with consultants, felt confidence and trust in them and (87%, 70/81) that the quality of information they received from staff was very good or excellent. The survey did not ask about pre-assessment procedures specifically but did reveal that patients needed more advice about leaving hospital. 77% (62/81) reported receiving written information at diagnosis about their condition or treatment but fewer reported this at outpatients (56%, 42/75) or after in-patient day case stays (60%, 32/53). 31% (15/49) did not receive but would have liked more information about financial or other benefits at hospital discharge.

### Receiving support

All EBCD patients were highly satisfied with the support they received throughout their journey, particularly from CNSs. All patients perceived CNSs to be the main source of support and emphasized their importance in making the journey easier, for example:

*" The best part of the service was definitely the breast care nurses. You might not speak to the same one all the time, but you know they’re there, and they will look after you, whatever the problem is."* (P7)

Patients also emphasized the importance of other support services in empowering them. A few patients felt these services, which included counseling, support groups and complimentary therapies, needed to be introduced earlier in the journey so that patients could make full use of them. The survey data similarly showed that patients had very positive experiences of care received from CNSs. Over 80% reported that they had received answers they could understand all or most of the time, had confidence and trust in them and were treated with dignity. Survey data provided no information on how useful patients found support services and simply reported 57% (43/75) patients were told about emotional support services, 63% (48/76) about complementary therapies and 72% (54/75) about a support group.

### Comparison of EBCD priorities and survey recommendations

In the EBCD process, day surgery was identified as a priority for improvement at the patient event and later voted as a key co-design area for improvement at the joint patient and staff event. The experience of being an in-patient was identified as a key ‘touchpoint’ for patients in the EBCD narratives, but was not identified as a co-design area for improvement. The survey report, however, recommended that the experiences of inpatient care, including day care needed to be improved in a number of specific areas including availability of nurses, pain relief, provision of information on treatment, possible side-effects of treatment and what to do after discharge and need for support at home. Neither EBCD nor the survey identified communication between departments or continuity of care as improvement priorities or recommendations. While the survey report recommended that organization of care across the whole pathway should be considered to match high levels of good experience around the diagnosis, the EBCD co-design improvement areas focused on the specific themes of the importance of communication between staff and patients, staff spending time with patients, written information, information about living with side-effects of treatment and about what happened afterwards. By contrast the survey recommendations focused on outpatient follow-up and treatment appointments suggesting the need to improve the appointment system, waiting times, the time patients have to ask questions, the written information routinely available to them and provided on other support available. Table 1 and 2 show the priorities for improvement and recommendations identified from each method (see Tables 1 and 2).

**Table 1:** EBCD priorities for improvement

	
** Day surgery:**	
• information about operating times/having to wait for hours	
• not being separated from friends/family too early	
• not feeling invisible/alone	
• not being rushed through recovery	
** Appointments:**	
• patients to receive letters on time, patients to be on lists and “not lost in the system”	
** Communication/ information along the way:**	
• importance of communication between staff and patients	
• staff spending enough time with patients	
• importance of written information	
• information about going through cancer treatment and living with the side effects	
• not feeling lost at end of treatment-more information about what happens next	
** Care at the end of treatment:**	
• patients to receive more information and support	

**Table 2:** Survey priorities for improvement


**Improve experience of in-patient stays (includes day care) to consider:**	
• availability of nurses	
• relief of pain and discomfort	
• provision of information on possible side-effects of treatment	
• written information on what to do after discharge	
• a record of treatment	
• discussion of needs for nursing or other support at home and benefits	
**Consider whether improvements in the organization of care across the whole pathway can be made to match high levels of good experience around time of diagnosis**	
**Improve experience of out-patient follow up and treatment appointments by considering:**	
• appointment system	
• waiting times	
• the time patients have to ask questions	
• the written information routinely available to them and provided on other kinds of support available	

## Discussion

This is the first study comparing EBCD patient narratives with postal surveys for the purpose of local service quality improvement, describing the priorities for improvement they identified for one breast cancer service. Our study would have been strengthened by comparing data from the second service (Hospital B) and also by using both methods with the same patients. Initially we had hoped to do this but there were unexpected delays in obtaining patient details for survey recruitment. Using the data we have, we first compare how each method informed our understanding of patients’ experiences and then discuss the implications for quality improvement approaches and research. We then draw out the key issues local managers need to consider in making the best use of both methods.

### How do the two methods inform our understanding of patients’ experiences of care?

Both the survey data and patient narratives identified some similar issues about patients’ experiences. For example, both datasets identified problems from the patient perspective with (a) how they moved through the system, (b) understanding what’s happening, and (c) receiving support. Both datasets identified similar issues with regards to *being an in-patient* although where patient narratives described negative experiences with one particular ward, the survey data were not sufficiently specific to pin point this. Similarly, narrative interviews provided more in-depth understanding (such as describing day surgery as being a fragmented service which patients felt was disorganized and chaotic, being separated from family and friends in the waiting area too early, and mixed sex facilities).

In many cases it was only by analyzing the open comments from the survey that we were able to identify the same or similar ‘problem’ areas to those in the patient narratives. The survey did not ask specifically or report separately about some areas such as day surgery, chemotherapy or radiotherapy, although some of the findings including patient comments might have suggested that further investigation was needed in these areas. Other areas such as waiting times, administrative problems and continuity of care were not asked about in the survey but emerged as ‘touchpoints’ in the patient narratives. A survey may therefore not cover all parts of the patient pathway and these areas could be expanded in future surveys. The fact that some are now covered by the English national survey, [22-24] suggests that the survey medium is not inevitably constricting but can be developed for quality improvement purposes. Examples of more structured programmes for the feedback of survey data and the development of improvement priorities have been developed in some US organizations [25].

Our comparative analysis suggests that in the absence of narrative accounts of patient experiences, better use should be made of open comments from survey data. By systematically analyzing these comments, issues raised in patient narratives were often identified. For example, three survey comments related to negative experiences during day surgery, thereby supporting the narrative interview findings. This suggests that survey data and specific open comments need to be fully analyzed to realize their full potential, not least because they may well shed light on specific aspects of a service where improvement work needs to be targeted. This does, however, raise a challenge for data analysts and quality improvement teams to decide how many comments are sufficient to justify investing time and effort in investigating a particular aspect of a service. One solution might be to increase the overall number of comments by inviting patients to comment after each section of a questionnaire rather than only at the end.

Furthermore, we suggest that open comments in survey responses should also be fed back to staff in the relevant services. The benefit of narrative interview data or other such qualitative approaches are that they can generate rich detailed descriptions of patient experience which are specific to a service. These narratives are inductive and are led by patients themselves, reflecting their experiences across the whole patient journey. They elicit those ‘touchpoints’ most important to them; that is, the emotional highs and lows of their experience. Surveys, on the other hand, represent a deductive approach with pre-determined fixed response questions which are useful when anonymous data are required and there is timely access to representative datasets for patient recruitment to surveys. They are better suited to making comparisons of patient experience between services and over time and to performance monitoring and benchmarking within and between organizations than a qualitative interview approach.

### What are the implications for quality improvement?

Achieving better understanding of patient experience can have a positive influence on health care by delivering services that patients, their families and carers need [16,23]. Insights into patient experience can potentially be used to improve patients’ ‘satisfaction’ with their care and their clinical outcomes [25-28]. Numerous studies have reported improvements subsequent to the systematic gathering of patient feedback by hospitals [12,14,17,29-39]. Despite such evidence, quality improvement based on patient experience has not been made a priority in many healthcare organizations, and few have adequate systems for co-coordinating the collection of such data, assessing its importance and implications and acting on the results in a systematic way [1,7].

Whilst the survey data did identify similar issues to the narrative ‘touchpoints’, the narratives provided more specific detail to enable potential service quality improvement (see Tables 1 and 2). There was also a difference in emphasis with the priorities arising from the narrative interviews placing greater emphasis on the ‘relational’ aspects of care as being those that mattered most to patients; that is care that “forms part of an on-going relationship with the patient and perhaps the family…” [40] (pp14) (for example, being separated from family and friends, feeling invisible/alone, communication with staff, time spent with staff). In contrast, the survey tended to highlight the more ‘functional’ aspects of care (for example, waiting times, availability of nurses, a record of treatment). Where relational views were covered by the survey, the results suggested more positive views about care, and ambiguity about whom patients wanted present at diagnosis.

We suggest that both approaches may have the potential to complement each other and we propose that future research tests the sequencing of these methods to include an initial preliminary survey that can capture important functional aspects of care, followed by an in-depth narrative-based analysis of the important relational aspects of patient experience. Gathering and analyzing interview and survey data from the same participants is also needed to understand any differing responses. Together these approaches could identify specific priorities for service improvement, while a planned change process (such as EBCD) could design and implement such improvements.

### Key issues for managers to consider when deciding on a local approach

Table 3 summarizes the strengths and weaknesses of the two approaches.

**Table 3:** Strengths and weaknesses of each method for local quality improvement

	**Strengths**	**Weaknesses**
**EBCD**	•covers whole patient pathway or journey •good for providing specific detail for local quality improvement purposes •engages clinicians and other staff •can be highly specific for a service •good on relational/emotional aspects of experiences •inductive: quality issues are determined by patients during the interviews and at patient events	•not always representative •generally thought to be relatively time-consuming and expensive when compared to surveys (although not the case in this study) •requires specific qualitative research skills to ensure a valid and reliable analysis •difficult to use for performance monitoring purposes over time or across institutions •requires sufficient participants for involvement in co-design group process.
**SURVEY**	•representative •can engage clinicians and other staff if fed back promptly and at service level •good for identifying issues with functional aspects of experience •may identify specific actions needed in some areas and other issues requiring further investigation •good for comparing between groups, institutions and for performance monitoring over time •open patient comments, if collected and analyzed, may provide additional understanding of issues identified	•may need to focus on specific service or parts of the patient journey to avoid burdening patients with a long questionnaire •findings may need further investigation to identify actions for local quality improvement purposes •deductive: quality issues are pre-determined by researchers/staff/patients in the development process •requires technical expertise around survey design, administration and analysis to ensure valid and reliable •relies on large enough sample size •Social desirability may influence telephone survey responses if they are not perceived as anonymous

We highlight three key issues that managers developing a local quality improvement programme should consider. These are the need for specific local data, the resource that is available for data collection, and investment in the change process adopted to develop priorities from data that lead to action.

National patient experience survey data have recently been analyzed and reported at an organizational (Trust) level for patients with the same diagnoses. For local quality improvement purposes, however, it is important to provide ward or clinic-level data to help ensure that points are specific enough to be acted on and feel ‘owned’ by staff in the services concerned. Providing such specific data is difficult to do in a national survey but easier in a local survey where questions can be adapted (as was the case in this study).

One criticism of EBCD and other narrative-based approaches is that they are time-consuming and relatively expensive. In this study one EBCD cycle in the breast cancer pathway took approximately 6 months’ work, involving a full-time researcher who recruited and interviewed patients and staff, analyzed data, compiled a patient film and fed back to both patients and staff. A half-time quality improvement facilitator was also required for that period (a total of 9 months of full time staff input). In addition, EBCD requires the time of patients and staff to be interviewed and to attend feedback events and co-design meetings (1.5-2 days per patient, and 1 day per staff member). By comparison, the survey used in this study required a full-time researcher working over 12 months to obtain agreement and relevant approvals, adapt and pilot the questionnaire, identify and obtain data on patients for the survey, de-duplicate hospital data, check patients addresses and vital status, distribute the survey with two reminders, and carry out the analysis. The contribution of hospital staff time to the survey included arranging a staff consultation about the survey by email, commenting on the questionnaire, identifying patients in clinic for pilot testing, and from hospital databases for the main survey. At least in this study, therefore, there was little difference in the time each approach took and the financial and staff time required. Given the rich and valuable material generated by the narratives, organizations may also wish to consider other less time-consuming qualitative methods including focus groups for gathering such data.

Finally a key difference between postal surveys and the patient narratives, as used within an EBCD project, is that the narratives are an integral part of an explicit change process that sets priorities for local quality improvement. If the (quantitative and open comment) results of postal surveys were used by staff and patients in this way, i.e. fed back and used to identify priorities for service improvement, the differences we noted between the two approaches may be reduced.

## Conclusion

Our first comparative analysis of EBCD patient narratives and patient experience survey data show that whilst survey data may act as a screening tool to identify problems, they do not always provide a full diagnosis of what to do to improve a service. Patient narratives, however, can delve into a problem and elicit important ‘clues’ to guide next steps for service improvement, as well as possible solutions. They can also be used within an EBCD process to inform and engage patients and staff in local quality improvement work. Our paper highlights the importance of the use of narratives in understanding and improving patient experience. These findings may have wider applicability in other services. Managers need to apply these survey and narrative methods carefully depending on the type of problem and quality improvement strategy required.

## Competing interests

The authors declare that they have no competing interests.

## Authors’ contributions

VT collected and analyzed the interview data, helped to interpret comparison with survey data and wrote the paper. JM helped to analyze and interpret the interview data and helped write the paper. TW helped design the study, collected and analyzed interview data, and helped write the paper. GR was consulted about study design, helped to interpret data and write the paper. PM collected and analyzed the survey data, helped to interpret the comparison, and revised the paper. AR conceived and helped design the study and revised the paper. MG helped to design and pilot the survey, interpret and feedback the results to staff and commented on the paper. ED conceived and helped design the study, helped to analyze and interpret the survey data and its comparison with the interview data, and helped write the paper. All authors read and approved the final manuscript.

## Pre-publication history

The pre-publication history for this paper can be accessed here:

http://www.biomedcentral.com/1472-6963/12/271/prepub
